# A Decade of Euroleague Basketball: an Analysis of Trends and Recent Rule Change Effects

**DOI:** 10.2478/hukin-2013-0058

**Published:** 2013-10-08

**Authors:** Erik Štrumbelj, Petar Vračar, Marko Robnik-Šikonja, Brane Dežman, Frane Erčulj

**Affiliations:** 1 Faculty of Computer and Information Science, University of Ljubljana, Slovenia.; 2 Faculty of Computer and Information Science, University of Ljubljana, Slovenia.; 3 Faculty of Computer and Information Science, University of Ljubljana, Slovenia.; 4 Faculty of Sport, University of Ljubljana, Slovenia.; 5 Faculty of Sport, University of Ljubljana, Slovenia.

**Keywords:** FIBA, basketball, statistics, three-point arc, shot-clock

## Abstract

The International Basketball Federation (FIBA) recently introduced major rule changes that came into effect with the 2010/11 season. Most notably, moving the three-point arc and changing the shot-clock. The purpose of this study was to investigate and quantify how these changes affect the game performance of top-level European basketball players. In order to better understand these changes, we also investigated past seasons and showed the presence of several trends, even in the absence of significant rule changes. A large set of game statistics for 10 seasons and 2198 Euroleague basketball games in which top European clubs competed was analyzed. Results show that the effects of the rule changes are contrary to trends in recent years.

## Introduction

Beginning with the 2010/11 season the Euroleague and several other European and most national basketball federations instituted new game rules. These changes, originally accepted by FIBA in 2008, include, among others, the addition of no-charge semicircles, moving the three-point arc 6.75 meters away from the basket, and changes to how and when the 24-second shot-clock is reset. It was assumed that these rule changes, the most significant in recent history, would have some effect on how the game of basketball is played. The goal of this paper was to investigate the effects of these changes using basketball game statistics. To that end, we analyzed the box-score statistics from the past 10 seasons of Euroleague competition, the highest level of European club competitions.

Based on the nature of rule changes, it was expected that the number and efficiency of two and three-point shots had been affected. In fact, this was not the first time that the distance of the three-point arc was changed in high-level basketball competition. In seasons 1994/95 through 1996/97 the NBA (National Basketball Association) three-point arc was moved closer to the basket and then back again. Also, since the 2008/09 season the NCAA (National Collegiate Athletic Association) basketball three-point arc has been moved farther away from the basket.

**Figure 1:**
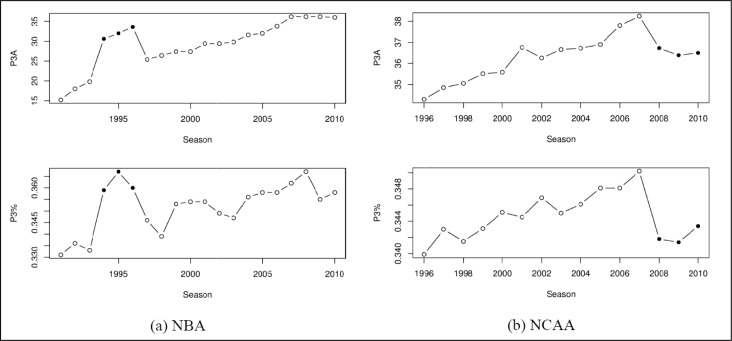
Total three-point attempts per game (P3A) and three-point shooting percentages (P3%) for past NBA and NCAA seasons

The NBA three-point arc is 23 feet 9 inches away from the basket (approximately 7.24 m). In Figure 1 it may be observed that seasons 1994/95 through 1996/97 are an exception (black points), as the three point arc was moved to 22 feet (approximately 6.70 meters) away from the basket. Since 1986, the NCAA three-point arc had been 19 feet 9 inches (approximately 6.00 m) away from the basket. For season 2008/09 and subsequent seasons it was moved to 20 feet 9 inches (approximately 6.32 m) away from the basket (black points). In both cases, moving the three-point arc farther away from the basket decreased the overall three-point percentage and reduced the number of three-point attempts. The same situation was expected to be observed in case of Euroleague.

Another major change is that the 24 s shot-clock in no longer reset for every foul or violation made by the defending team. If the throw-in is to be administered in the frontcourt, the shot-clock will only be reset if there are 13 or fewer seconds remaining and it will only be reset to 14 s instead of 24. This reduces the average time per ball possession and should increase the number of ball possessions per game. Therefore, in terms of possessions, the game pace is expected to increase and, if that is indeed the case, the decrease in three-point attempts should result in an increase of two-point attempts, an increase in turnovers, or both.

Some of these hypothesized changes are contrary to trends in recent years in which the number of possessions was decreasing and the number of three-point attempts was increasing. While these trends are generally speculated to be true, there is, as far as the authors are aware, no published statistical analysis of this topic. Therefore, prior to comparing the changes between 2009/10 and 2010/11, we also analyzed the trends in previous seasons.

Overall, there is little research that addresses the rule changes in basketball. Podmenik et al. (2012) analyzed shot accuracy of female basketball players before and after a smaller and ligher basketball was introduced in the 2004/05 season (a size 6 ball was introduced instead of a size 7). They did not find any significant differences in field-goal accuracy, but found a significant decrease in free-throw accuracy. The kinematics of basketball shooting were studied by Miller and Bartlet (1993, 1996) and Tang and Shung (2005). They showed that shot kinematics change with distance as does the role of individual muscles. As expected, their experiments also showed that shooting accuracy decreases with increasing distance from the basket. Cormery et al. (2008) studied the physiological characteristics of basketball players in the years before and after the 2000 rule change, when the shot-clock was changed from 30 to 24 s, effectively increasing the pace. Lilly (1987) showed how the average number of points per game increased when a 45 s shot-clock was introduced in men’s college basketball in the US. The author observed an increased level of fitness and concluded that the rule change was a contributing factor. Erčulj et al. (2003) also studied the effects of this change on the 2000 (before) and 2001 (after the change) Men’s Junior European Championships. After the change, the number of possessions and points per game increased (for a survery of sport rule modification studies see Arias et al., 2011). Other statistics-based basketball research mostly focuses on analyzing home-team advantage (Pollard and Pollard, 2005; Gomez and Pollard, 2011; Tauer et al., 2009), the effect of different factors on basketball players’ performance (Sampaio et al., 2010) and using statistics to discriminate between winning and losing teams (Lorenzo et al., 2010).

## Material and Methods

A set of basketball statistics from Euroleague basketball between 2001 and 2010, for a total of 2198 games, was compiled. The following notation for standard basketball statistics throughout the paper was used: PTS - points scored, P2A - two-point attempts, P2% - 2pt percentage, P3A - three-point attempts, P3% - three-point percentage, FTA - free throw attempts, FT% - free throw percentage, TR - total rebounds, AS - assists, ST - steals, TO - turnovers, B - blocked shots, FC - personal fouls committed, FD - personal fouls drawn, and NP - number of possessions. Unless otherwise noted, we used game totals (that is, the sum of both teams’ statistics). A summary of seasonal averages is presented in Table 1.

For the purposes of this paper, the following definition of the number of possessions was used: NP = P2A + P3A + ½ FTA + TO (Erčulj et al., 2003). Note that some definitions of a basketball possession count two (or more) consecutive possessions due to an offensive rebound as a single possession (Kubatko et al., 2007). The number of possessions is considered an indicator of the tactical pace of a game, as opposed to the physical pace of a game, which would require measuring the players’ activities or physiological variables such as heart rate or lactate.

The statistical analysis was performed in two parts. First, we analysed the trends, and, second, the difference between the last season before the major rules changes and the first season after the rules changes. Analysis of trends included preliminary ANOVA tests for each game statistic separately to exclude statistics where no season was significantly different from the others. After visual inspection we assumed linear trends and fit linear models to test the significance of the trends. Welch two-sample tests for comparing the last season before the rule change and the first season after the rule change were used. Made/missed shots of a particular type are assumed to follow a binomial distribution, so a Z-test was used to test the equality of the proportions of success. Finally, our results were independently verified by and discussed with Euroleague coach Sašo Filipovski.

## Results

**Figure 2:**
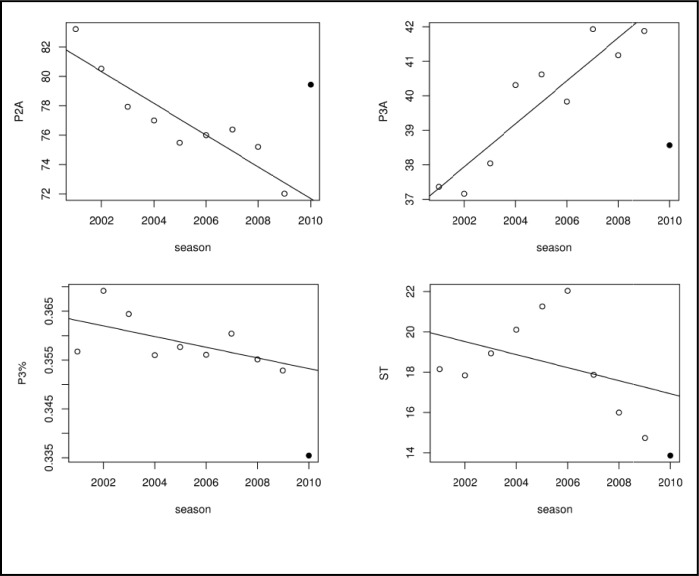
Seasonal averages for two-point and three-point attempts, three-point percantage and steals per game.

Preliminary analysis of variance revealed that there are significant differences between seasons for the period between 2001/02 and 2009/10 for all variables (all p values < 0.001) with the exception of the average three-point shooting percentage (p = 0.4605). Further visual inspection (see Figure 2) of two-point and three-point attempts per game suggested a linear trend. The number of three-point attempts was increasing, while the number of two-point attempts was decreasing. The 2010/11 season is an exception to this trend - the number of three-point attempts was decreasing and the number of two-point attempts was increasing. The three-point percentage was decreasing during the period from 2001/02 to 2009/10. However, the 2010/11 three-point percentage was smaller than in previous seasons.

Detailed results of the subsequent regression analysis of the trends are shown in Table 2. The previously mentioned trends for the number of two-point and three-point attempts are statistically significant. At approximately 1.1 two-point attempts fewer per game each season and 0.6 three-point attempts more per game each season, they are also statistically significant. Between-season differences in three-point shooting percentage were rejected in the preliminary analysis, but we still reported the results for the trend, which was not statistically significant. Other significant trends included a decreasing number of possessions and number of points per game, a decreasing number of fouls and free throw attempts, an increasing number of blocked shots, and an increasing number of assists. The number of steals per game followed a non-linear non-monotonic trend (see Figure 2). The number of steals per game was increasing throughout the 2001/02 – 2006/07 period, followed by a substantial drop in the 2007/08 season and a further decrease in subsequent seasons.

The results for the analysis of the differences between 2009/10 (the last season before the rule changes) and the sample of games from 2010/11 (the first season after the rule changes) are shown on the right-hand side of Table 2. Two-point shooting effectiveness decreased from 52.69% to 50.75% (p = 1.10 • 10-3) and 3-point shooting effectiveness decreased from 35.29% to 33.54% (p = 2.44 • 10-2). The change in free-throw shooting percentage from 73.8 to 74.1 was not found to be significant (p = 0.64).

In accordance with our initial expectations and against recent trends, the number and efficiency of three-point shots decreased. The expected increase in the number of possessions implies that the number of two-point shots increased (due to fewer three-point shots). Indeed, differences were found to be significant for both variables. A decrease in shooting percentages explains the increase in total rebounds. The significantly lower number of personal fouls and free throws is in accordance with the trends in recent years. The drop in two-point shooting percentage was also found to be significant.

## Discussion

In the last decade, the game of basketball has, through increased training and improved tactical knowledge, undergone a process of rationalization. The passing, shooting, and shot-selection have become more efficient. Players are more patient in setting up offensive opportunities, passing frequency has increased, and there are more on-ball screens (pick-and-roll plays, etc...). These explain both the increasing number of assisted shots and a decreasing number of shots in general. However, it is noteworthy that the decreasing tactical tempo (the total of offensive opportunities and turnovers) does not imply that the physical tempo, in terms of speed and explosiveness of movement, has also decreased. The number of blocked shots has also been increasing. While the absolute change in the number of blocked shots is small when compared to other variables, it should be taken into account that a blocked shot is a less frequent event. Relative to the average number of such events, the change is substantial and larger than changes in most other variables.

The new rules also brought changes to how the shot-clock is reset. The shot-clock is set to 14 seconds instead of 24 if there are 14 or fewer seconds left on the shot clock at the time of a violation or non-shooting personal foul by the defending team. With at most 32 non-shooting fouls per game and few other such violations, the effects of this change are much smaller than when the shot-clock was reduced from 30 to 24 s in 2000. Compared to 2009/10, the number of possessions and points increased in 2010/11, but the differences are not as large as those observed after the 2000 change (Erčulj et al., 2003) or the introduction of the 45 s shot-clock in the NCAA (Lilly, 1987). The question, whether this minor change would also result in an increased fitness of basketball players, as the 2000 change did (Cormery et al., 2008), remains open.

The usage of personal fouls has become more rational, with a decreasing share of irrational or reckless fouls and increasing share of intentional tactical fouls to break up fast-breaks and prevent the opponent from scoring easy points. This, together with a decreasing number of possessions and better officiating that allows cleaner play with little illegal contact, has resulted in a decreasing number of personal fouls.

It was hypothesized that the main reasons behind the drop in two-point shooting percentage were an increased frequency of and larger area for two-point shots - some shots, which were considered three-point shots, are now further away from the basket. The available statistical data do not allow further investigation of reasons behind this change, so we delegated this to further work. Also, a decrease in three-point accuracy can be explained by an increased distance of three-point shots. However, the statistical data do not allow us to investigate if a part of a decrease in accuracy is due to changes in players’ shot kinematics to account for an increase in distance. Shot kinematics change with shot distance (Miller and Bartlet, 1993, 1996) and different training might be more appropriate after the change in distance (Tang and Shung, 2005). Therefore, in future seasons, three-point accuracy might increase as players and training regimes are modified to account for the changes.

With the absence of any major rule changes during the 2001/02 – 2009/10 period, it was assumed that trends during this period, if any existed, would be linear or at least monotonic. The number of steals per game did not follow this trend, so additional research was necessary. No rule changes was put into effect during the 2007/08 season that would explain the abrupt change in steals per game. However, during that period an Officiating criteria memorandum was accepted by ULEB (Union of European Leagues of Basketball) that would also affect the Euroleague (www.euroleague.net/news/i/16729/180/item).

The motivation behind the officiating changes was to eliminate illegal contacts that prevent open and skillful play. Special attention was given to the elimination of illegal use of hands, forearms, and legs during post play and illegal use of hands or forearms by a defensive player when facing the opponent. Taking these options away from the defending player would explain the drop in the number of steals. Furthermore, the coaches and referees anticipated that players would quickly adapt to this new refereeing criteria, so an increase in the number of personal fouls was not a concern. This was indeed the case, as the number of personal fouls per game decreased (Table 1, season 2007/08).

To conclude, the Euroleague data confirm our initial hypotheses that increasingly frequent three-point shooting was set back by moving the arc farther away from the basket, and that the number of two-point attempts increased. The same type of changes was observed when the three-point arc was moved in the NBA and the NCAA. Furthermore, the 2010/11 FIBA rule changes have resulted in a higher number of possessions and points per game. Increasing the number of points scored and speeding-up an otherwise slowing-down pace of the game is arguably a desired effect, making the games more interesting for the audience. We also found an abrupt trend reversal in the number of steals per game that can be explained by changes in refereeing criteria.

## Figures and Tables

**Table 1:** Number of games and average values for 10 seasons of the Euroleague

	N	PTS	P2A	PT%	P3A	P3%	FTA	FT%	TR	AS	ST	TO	B	FC	NP
2001/02	274	161.5	83.0	0.521	37.4	0.357	48.7	0.722	64.0	24.2	18.1	28.4	4.8	48.3	173.1
2002/03	220	157.0	80.4	0.517	37.2	0.369	45.2	0.724	63.2	24.4	17.8	25.8	4.8	46.0	166.0
2003/04	219	157.8	77.8	0.531	38.0	0.364	45.9	0.733	62.6	26.1	18.9	25.7	4.5	46.1	164.5
2004/05	228	155.9	77.0	0.519	40.3	0.356	45.2	0.729	64.4	25.6	20.1	26.9	5.0	45.8	166.7
2005/06	231	149.9	75.1	0.505	40.6	0.358	42.6	0.712	65.2	25.1	21.3	27.6	4.9	43.4	164.7
2006/07	230	152.4	75.7	0.516	39.8	0.356	43.6	0.727	64.8	25.7	22.0	28.4	4.9	44.3	165.8
2007/08	231	154.1	75.8	0.524	41.9	0.360	40.0	0.733	63.2	25.8	17.9	27.2	5.3	41.9	165.0
2008/09	188	152.3	75.2	0.518	41.2	0.355	40.7	0.749	64.4	25.0	16.0	27.5	5.3	42.2	164.3
2009/10	188	150.9	72.0	0.527	41.9	0.353	41.6	0.738	64.1	27.7	14.7	28.2	5.3	43.0	162.9
2010/11	189	148.5	79.4	0.508	38.6	0.335	39.2	0.741	67.7	27.5	13.9	27.8	5.3	42.6	165.4

**Table 2:** Results of the regression analysis of the trends and the Welch two sample test comparison of the last two seasons. Regression coefficient, standard error and t-value for regression of trends and t-value and p-value for Welch two sample test. Results significant at the level of 95% are underlined.

	Seasons 2001/02 – 2009/10				2009/10 vs 2010/11	

	Coeff.	s	t	p	t	p
PTS	−1.127	0.292	−3.853	6.27 · 10^−3^	1.459	1.45 · 10^−1^
P2A	−1.078	0.175	−6.169	4.59 · 10^−4^	−8.080	9.00 · 10^−15^
P2%	0.000	0.001	0.184	8.59 · 10^−1^	3.172	1.64 · 10^−3^
P3A	0.624	0.102	6.115	4.84 · 10^−4^	4.871	1.65 · 10^−6^
P3%	−0.001	0.001	−1.898	9.94 · 10^−2^	2.763	6.00 · 10^−3^
FTA	−0.924	0.167	−5.534	8.74 · 10^−4^	2.064	3.97 · 10^−2^
FT%	0.003	0.001	2.294	5.55 · 10^−2^	−0.054	9.56· 10^−1^
TR	0.093	0.112	0.827	4.35 · 10^−1^	−4.391	1.47 · 10^−5^
AS	0.257	0.107	2.391	4.81 · 10^−2^	0.430	6.67 · 10^−1^
ST	−0.323	0.301	−1.075	3.18 · 10^−1^	1.935	5.38 · 10^−2^
TO	0.153	0.130	1.172	2.79 · 10^−1^	0.708	4.80 · 10^−1^
BF	0.084	0.022	3.875	6.09 · 10^−3^	0.047	9.63 · 10^−1^
FC	−0.707	0.122	−5.789	6.71 · 10^−4^	0.586	5.58 · 10^−1^
NP	−0.763	0.281	−2.718	2.99 · 10^−2^	−2.239	2.57 · 10^−2^
